# Movement patterns of invasive red swamp crayfish vary with sex and environmental factors

**DOI:** 10.1038/s41598-025-96379-8

**Published:** 2025-04-24

**Authors:** Maggie Raboin, Brian M. Roth, Aaron Sullivan, Ann L. Allert, Jim A. Stoeckel, Lucas R. Nathan, Kathleen B. Quebedeaux, Matthew D. Sholtis, Justin R. Smerud, Richard A. Erickson, Aaron R. Cupp

**Affiliations:** 1 Western Fisheries Research Center, Columbia River Research Laboratory, U.S. Geological Survey, 5501A Cook-Underwood Rd, Cook, WA 98605 USA; 2 Department of Wildlife and Fisheries, Michigan State University, East Lansing, MI 48824 USA; 3 Columbia Environmental Research Center, U.S. Geological Survey, 4200 New Haven Road, Columbia, MO 65201 USA; 4 School of Fisheries, Aquaculture, and Aquatic Sciences, Auburn University, Auburn, AL 36849 USA; 5 Michigan Department of Natural Resources, Lansing, MI 48933 USA; 6 Upper Midwest Environmental Sciences Center, U.S. Geological Survey, 2630 Fanta Reed Rd., La Crosse, WI 54603 USA

**Keywords:** Movement ecology, Invasive species, Freshwater ecology, Acoustic telemetry, Hidden Markov models, wAKDE, Freshwater ecology, Invasive species

## Abstract

Invasive species disproportionately invade freshwater ecosystems, threatening biodiversity. Defining when, where, and why aquatic invasive species move can help inform management strategies, yet the movement ecology of some of the most pervasive invasive species remains unknown. Red swamp crayfish (*Procambarus clarkii*; RSC) are the most widespread invasive crayfish and negatively affect ecosystems worldwide. We employed high-dimensional acoustic telemetry to investigate the movement patterns of 24 individual RSC across three months in an invaded water body. We assessed the effects of various extrinsic factors, such as time of day, temperature, precipitation, and proximity to the water’s edge, along with intrinsic factors, such as sex, reproductive form, and size, at different scales, including movement steps, range distribution, and behavioral states. We found that movement patterns across all scales were overwhelmingly driven by sex and reproductive form. Furthermore, RSC showed increased overall activity at night and near the water’s edge. By establishing baseline movement patterns and identifying key contributing factors, these findings provide a foundation for the development of adaptive management strategies for controlling invasive RSC populations.

**Supplementary Information:**

The online version contains supplementary material available at 10.1038/s41598-025-96379-8.

## Introduction

Freshwater ecosystems make up less than 2% of Earth’s surface yet are home to nearly 10% of global animal diversity^1,2^. Yearly declines in freshwater species are consistently greater than declines in terrestrial species, making freshwater ecosystems some of the most vulnerable on Earth^3^. One major threat to freshwater biodiversity is the prevalence of invasive species, which disproportionately invade freshwater ecosystems^4,5^. Despite the importance of freshwater ecosystems to global biodiversity and their vulnerability to invasive species, little is known about the movement patterns of invasive freshwater animals. Technological advances in tools used to track aquatic animals, such as acoustic telemetry, allow for increasingly fine-scale positioning data that can be used to define when, where, and why invasive species move^6–8^. Understanding the movement ecology of invasive species could help characterize the impact of these species on ecosystems and improve the effectiveness of management strategies by enabling targeted control actions that are flexible in time and space^6,9^.

An individual animal moves through space over a lifetime in a series of movement steps (displacement between successive positions) that can be grouped into movement phases representing behavioral states such as foraging, mate-searching, or resting^10^. Movement patterns at different spatial and temporal scales may be driven by intrinsic and extrinsic factors that operate differently depending on the scale of movement^10,11^. For example, an animal’s movement over minutes may be driven by food availability whereas their movement over months may be driven by reproductive status. For invasive species, understanding drivers of animal movement and the scales at which they operate could illuminate dispersal dynamics^11^, invasion pathways^12^, habitat preferences^12,13^, or barrier design^14^, and highlight key individuals in a population that differentially influence these aspects^15,16^. Additionally, establishing baseline movement patterns and contributing factors for an invasive species can illuminate patterns that may be exploited for effective control or eradication, and as a reference for assessing the effectiveness of subsequent interventions^14,17^.

Fundamental questions remain concerning the movement patterns of some of the most widely distributed invasive animals. This is particularly true for invasive aquatic animals, such as red swamp crayfish (*Procambarus clarkii* Girard, 1852). Red swamp crayfish (hereafter, RSC) are native to northern Mexico and the southern United States but have been introduced to all continents except Antarctica and Oceania^18^. They possess high growth rates, broad abiotic tolerances, and omnivorous feeding habits – traits that favor their establishment in new habitats^19,20^. Red swamp crayfish can greatly alter ecosystem processes and decrease water quality in invaded water bodies through macrophyte consumption^21^, propensity to spread disease to native crayfish (e.g., crayfish plague *Aphanomyces astaci*)^22^, and extensive burrowing activity^23^. Currently, control efforts in areas with invasive RSC include a variety of approaches from mechanical removal to chemical treatment^24–26^.

Effective application of treatments and success of management strategies depend, in part, on an understanding of RSC movement patterns. Previous research with radio-telemetry data revealed substantial inter-individual variation in RSC movement, but a general tendency among all individuals toward nocturnal activity^27–29^. However, individual RSC may still move long distances during the day^28,29^. In addition, multiple studies suggest that RSC travel in bouts of long distance (exploratory) and short distance (encamped) movements^27–29^. Beyond these trends, researchers have been unable to find consistent factors contributing to RSC movement patterns, but sex, size, reproductive form, and seasonality may contribute to movement patterns at different spatiotemporal scales^27–29^. Traditional radio-telemetry systems are limited in the frequency of location fixes, range, and tracking duration, leaving researchers to infer behavior over large spatial and temporal gaps between relocations. In contrast, acoustic telemetry allows for the collection of fine-scale positional data of many individuals over long distances and periods of time^6,7^. Acoustic telemetry is relatively affordable and has become one of the most common methods of tracking aquatic animals worldwide^30^. However, it has not been widely applied to study the movement ecology of invertebrates; an important data gap to better understand the invasion success of prolific species, such as RSC.

This study used acoustic telemetry to understand the extrinsic and intrinsic factors that may contribute to RSC movement patterns in their invasive range. We quantified the movement of invasive RSC across three spatiotemporal scales to address fundamental gaps in our understanding of their movement patterns. Specifically, we used multiple analytical frameworks to examine the effects of predictors on RSC movement steps, range distribution, and behavioral states. We considered extrinsic factors such as hourly temperature, precipitation, and distance to the water’s edge as well as intrinsic factors such as sex, reproductive form, and size of individual crayfish. We expected that RSC movement patterns would align with findings from previous radio-telemetry studies but that with the greater spatiotemporal resolution of acoustic telemetry, we would also find meaningful effects of individual-level and environmental attributes on movement patterns across scales. Our results describe the movement ecology of invasive RSC and have broad implications to better understand how movement patterns contribute to invasion success and might be exploited for effective management.

## Results

### Movement steps

**Fig. 1:**
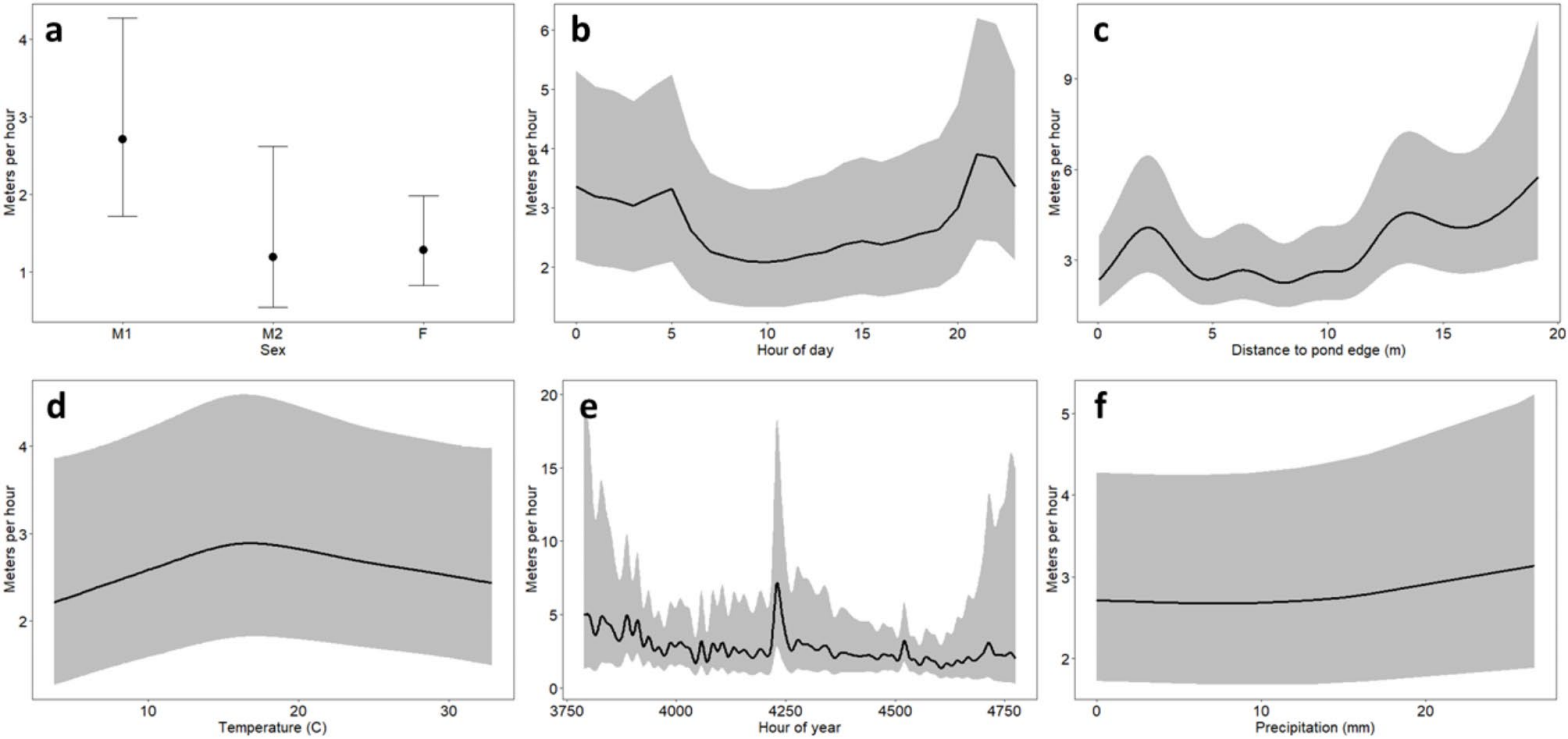
Estimated relationships between (**a**) sex, (**b**) hour of day, (**c**) distance to pond edge (m), (**d**) temperature (°C), (**e**) hour of year, (**f**) precipitation (mm) and movement steps (distance per hour) analyzed with a generalized additive model (GAM) from invasive red swamp crayfish (*Procambarus clarkii*) spatial locations May–July 2021. Error bars and ribbons represent 95% confidence intervals. All relationships contributed to variation in movement steps except for precipitation.

Movement steps ranged from 0.01 m to 100.18 m per hour (mh^− 1^) with a mean of 1.99 mh^− 1^ (standard deviation [SD] = 4.72 m, *N* = 14,845)^31^. The full generalized additive model (GLM) explained 54.9% of the deviance in movement steps. All covariates except hourly precipitation contributed to variation in movement steps (Table S1). Red swamp crayfish individuals varied considerably in their movement steps through time (*p* < 0.001). Females and nonreproductive males (M2) were more similar in their movement steps (mean = 1.51 m, SD = 3.41 m and mean = 1.31 m, SD = 1.39 m, respectively) than they were to reproductive males (M1) (mean = 2.84 m, SD = 6.45 m; *p* = 0.010); however, confidence intervals of movement steps were overlapping for all sexes (Fig. 1a). RSC movement steps differed over the course of a day (*p* < 0.001), with longer movement steps occurring at night (19:00–06:00) and shorter movement steps occurring during the day (06:00–19:00) indicating nocturnal activity (Fig. 1b). Movement steps differed with distance to pond edge with RSC movement steps reaching peaks around 2.5 m and at distances greater than 12 m from the pond edge (Fig. 1c). Air temperature influenced movement steps (*p* = 0.006) with longer movement steps occurring between 15 and18°C (Fig. 1d). Hour of year contributed to RSC movement steps (*p* < 0.001), suggesting variation in crayfish movement throughout the study period. This relationship showed a rapid increase in RSC movement steps centered around hour of year 4223 (6/25/21–/26/21) corresponding with a major rainstorm and the maximum precipitation measures in our data set (hourly precipitation max = 26.61 mm) (Fig. 1e). Despite this event, we did not find that precipitation throughout our study period contributed to RSC movement steps (Fig. 1f).

### Range distribution

**Fig. 2:**
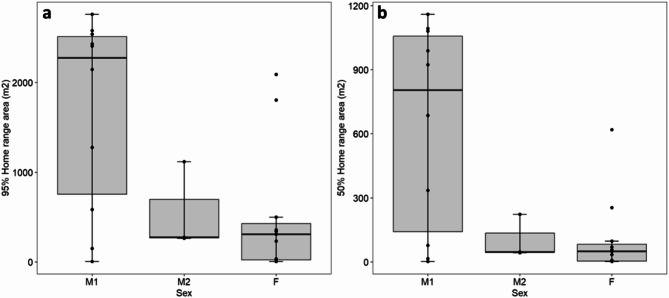
Estimated (**a**) 95% and (**b**) 50% range distributions for invasive red swamp crayfish (*Procambarus clarkii*) by sex. Boxes represent the interquartile range for each distribution of values for each group; horizontal line within each box represents the median value; vertical lines extend to the most extreme values within 1.5 interquartile range of the 25th and 75th percentile of each group.

Range distributions (weighted autocorrelated density estimates [wAKDEs]) ranged from 4.46 m^2^ to 2761.56 m^2^ at the 95% level, with a mean of 1009.49 m^2^ (SD = 1026.55 m^2^) and from 1.53 m^2^ to 1160.03 m^2^ at the 50% level, with a mean of 327.84 m^2^ (SD = 420.80 m^2^). For both 95% and 50% range distribution estimates, the generalized linear model (GLM) with the lowest Akaike’s Information Criteria [AICc] was that with sex alone as the predictor variable (Table S2). At the 95% range distribution level, range distribution estimates of females and M2 males were smaller and more similar to one another (mean = 523.24 m^2^, confidence interval [CI] = 202.54–1333.41 m^2^ and mean = 556.99 m^2^, CI = 151.91–2920.00 m^2^, respectively) than to M1 males (mean = 1687.86 m^2^, CI = 917.23–3628.20 m^2^), but confidence intervals of estimates for all sexes were overlapping (Fig. 2a, Table S3). Similarly, at the 50% range distribution level, range distribution estimates of females and M2 males were smaller and more similar to one another (mean = 108.16 m^2^, CI = 31.81–337.20 m^2^ and mean = 101.80 m^2^, CI = 25.45–827.10 m^2^, respectively) than to M1 males (mean = 636.23 m^2^, CI = 311.39–1623.93 m^2^), but confidence intervals of estimates for all sexes were overlapping (Fig. 2b, Table S3).

### Hidden Markov modeling

We determined via AIC model selection that a two-state hidden Markov model [HMM] with covariate effects of sex, time of day, and distance to the pond edge best fit the data (Table S4; Fig. S1 for diagnostic plots). The HMM identified and characterized RSC estimated positions into two behavioral states that differed in their step lengths and turning angles and aligned with previous descriptions of RSC movement^27,29^. The HMM described an encamped state, characterized by a short step length with crayfish moving a mean of 0.40 m (SD = 0.29) every hour and a mean turning angle of -3.14 radians (concentration = 0.29), and an exploratory state, characterized by a long step length with crayfish moving a mean of 3.15 m (SD = 6.23) every hour and a mean turning angle of 0 radians (concentration = 0.70) indicating more directionally persistent movement than those in the encamped state (Fig. S2).

**Fig. 3:**
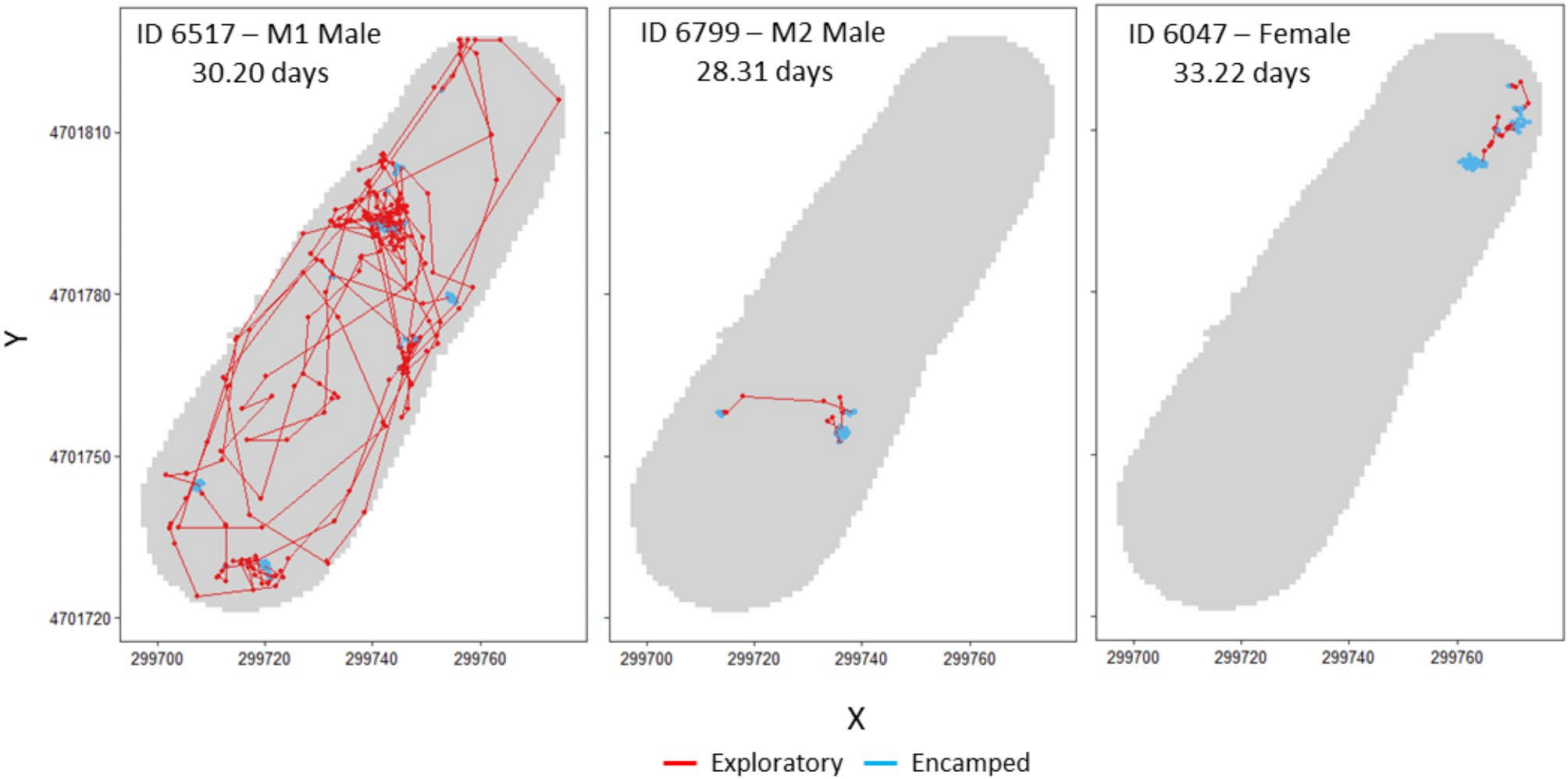
Example paths of three individual red swamp crayfish (*Procambarus clarkii*), colored by behavioral state. Data used to estimate paths and behavioral states were collected over a similar length of time (mean = 30.58, SD = 2.48) and overlapping time periods (5/28/21–7/19/21).

**Fig. 4:**
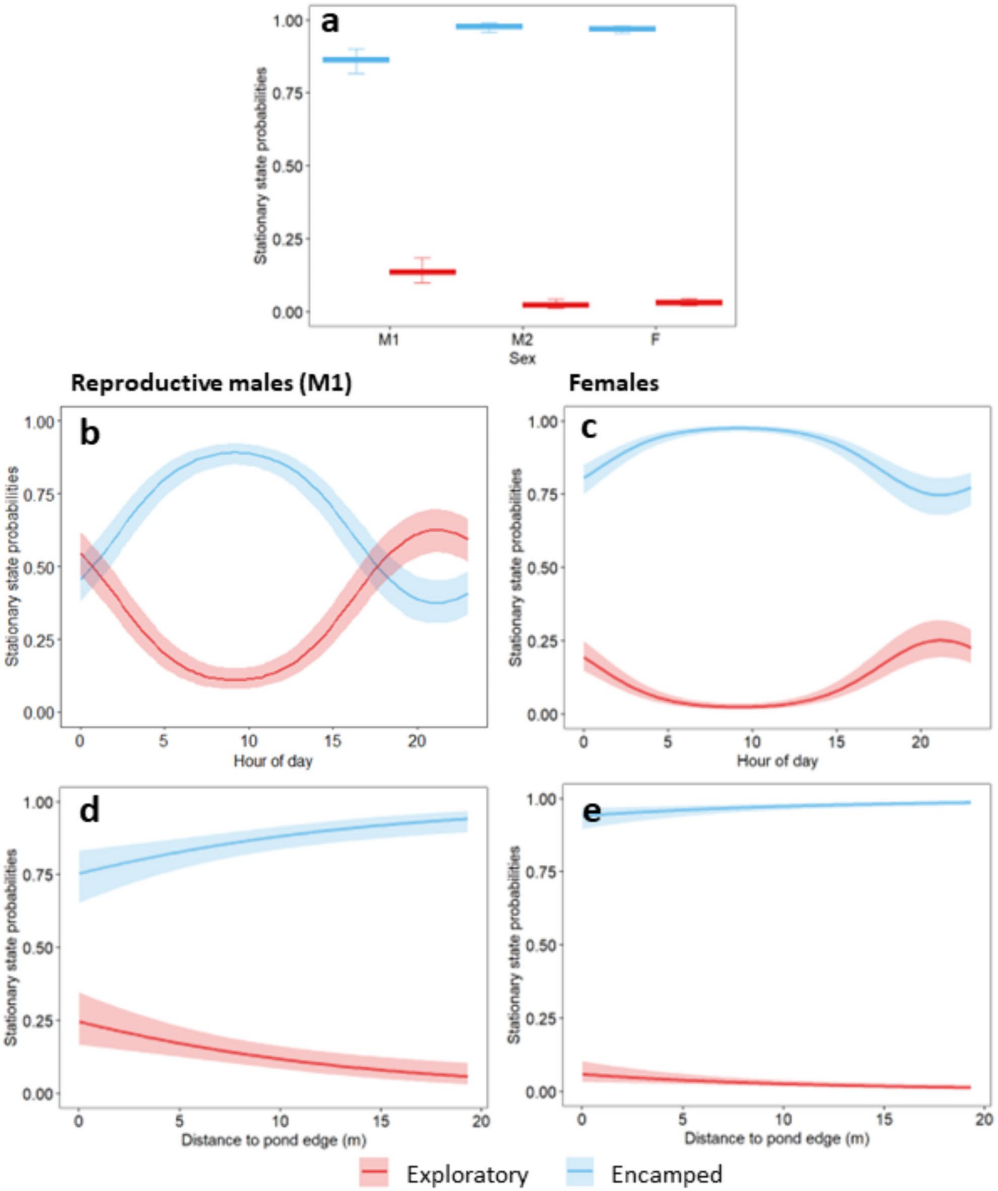
Stationary state probabilities for red swamp crayfish (*Procambarus clarkii*; RSC) with 2 behavioral states (encamped, exploratory). Stationary state probabilities were calculated using mean covariate values of 11:34 (hour of day) and 8.12 m (distance to pond edge). Figures on the left show stationary stable probabilities for M1 reproductive male RSC and figures on the right show stationary stable probabilities for female RSC. Lines show model estimates across covariate values, and boxplot whiskers and shading show 95% confidence intervals.

Overall, RSC spent 79.0% of the time in an encamped state and 21.0% of the time in an exploratory state (total = 16,210 1-hour locations) (Fig. 3). State probabilities differed across the range of covariates measured with M1 males having a higher probability of being in an exploratory state than M2 males or females (Fig. 4a). For example, the mean proportion of time spent in an exploratory state was 37.9% (SD = 26.4%, *N* = 10) for M1 males, 11.8% (SD = 11.0%, *N* = 11) for females, and 6.4% (SD = 4.2%, *N* = 3) for M2 males. The effects of hour of day and distance to the pond edge on state probabilities differed between sexes such that M1 males had a higher probability of being in an exploratory state than an encamped state during the evening and a slight increase in their probability of being in an exploratory state when they were closer to the pond edge, whereas females had a higher probability of being in an encamped state than an exploratory state at all times of day and all distances from the pond edge (Fig. 4b-e). Nevertheless, females did have slight increases in their probabilities of being in an exploratory state during the evening and near the pond edge (Fig. 4b, d).

**Fig. 5:**
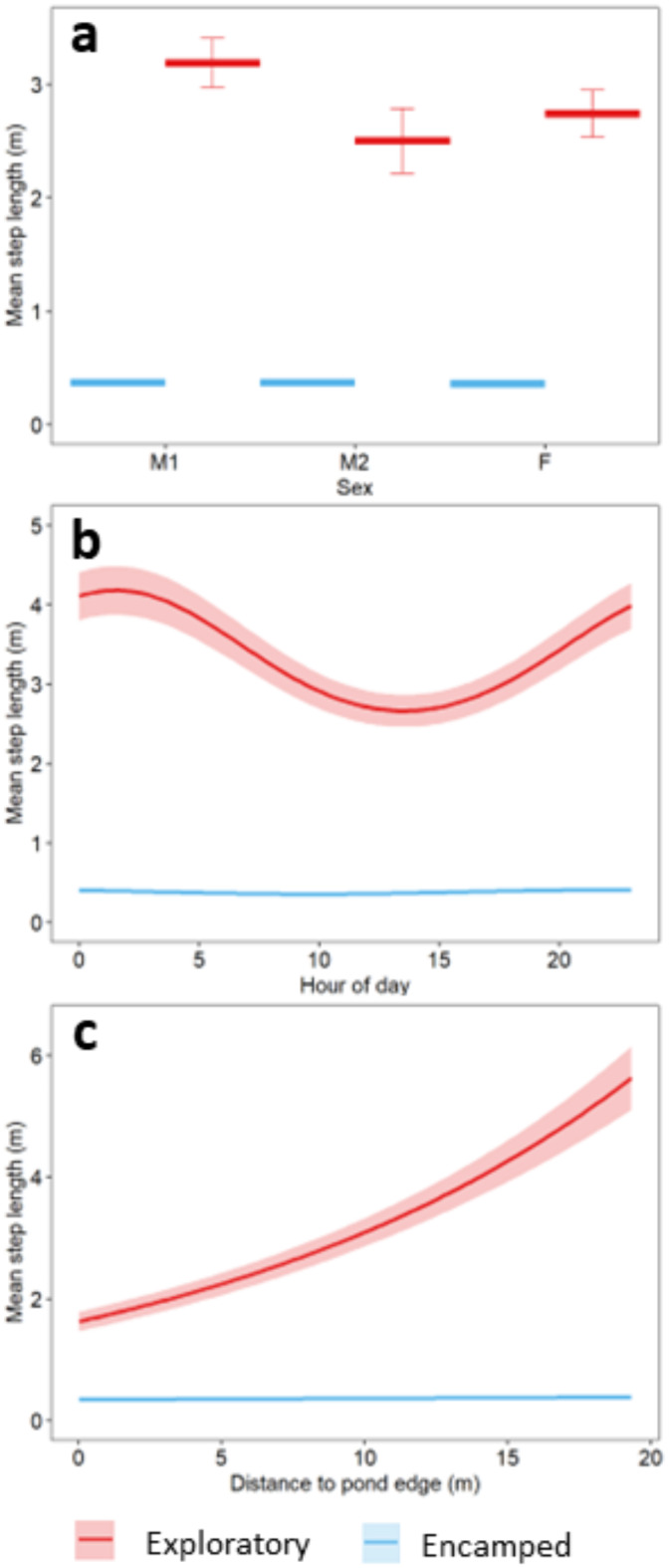
Movement parameter estimates of step length across (**a**) sex, (**b**) hour of day, and (**c**) distance to pond edge for all red swamp crayfish (*Procambarus clarkii*). Lines show model estimates across covariate values, and boxplot whiskers and shading show 95% confidence intervals.

Mean step length for RSC in each behavioral state varied with modeled covariates in ways that aligned with our findings on movement steps and range distribution estimates. Overall, covariates had a stronger measured effect on mean step length when RSC were in the exploratory state than when RSC were in the encamped state (Fig. 5). While in an exploratory state, mean step lengths for M1 males were longer than those of females and M2 males, were shortest during the day and longest at night and were longer further away from the pond edge (Fig. 5).

## Discussion

Invasive RSC are some of the most globally widespread invasive freshwater animals and yet, little is known about their movement ecology^18,20^. By combining acoustic telemetry with various analytical techniques, we were able to create a detailed picture of RSC movement patterns and their contributing factors. We found that RSC movement patterns were primarily driven by intrinsic factors, such as sex and reproductive form, but were also influenced by extrinsic factors, such as time of day, temperature, and distance to the pond edge. Our results establish a baseline understanding of RSC movement patterns that can be used as a model for further exploration of RSC movement ecology in different invasion, habitat, and management contexts.

Sex was the most important contributing factor to variation in all measures of movement. While our analyses suggest that reproductive males had larger movement steps, spent more time in an exploratory state, and had larger range distributions than females and M2 males, we were only able to find a clear difference between sexes in their probability of being in an exploratory state. This is likely due in part, to a limited sample size, especially of non-reproductive males (*N* = 3), in our study. Our study took place during a time when RSC are expected to be reproductively active^32,33^. The movement patterns we found are consistent with a scramble competition mating system where males compete, in part, through increased movement which results in greater encounter rates with receptive females^34,35^. Sex differences in movement patterns may result in sex-biased control efforts depending on how management actions such as trapping or pesticide treatments are administered. Studies have frequently reported male-biased trapping of RSC and other crayfish species, with some exceptions^24,36–38^. Our results suggest that trapping efforts targeting female RSC during the reproductive period might include more traps that are tightly spaced to account for lower movement rates among females. Further research would be beneficial to understand sex-differences in RSC movement across seasons.

Crayfish movement patterns were partly driven by time of day and distance to the pond edge. The results of our combined analytical methods – movement steps and behavioral states – reveal that while RSC moved more at night overall, they still moved a considerable amount during the day (Fig. 1b). This finding aligns with previous research^39,40^. Additionally, we found that the probability that RSC were in an exploratory state was highest in the evening, and for M1 males this probability was even greater than being in an encamped state (Fig. 4b, c). These findings suggest that trap encounters for reproductive males may be highest during the evening, and sex-bias in trapping may be exacerbated if limited to overnight hours. Finally, our findings show that all crayfish were more likely to be in an exploratory state near the edge of the pond (Fig. 4d, e) and that RSC had larger movement steps near the edge and center of the pond (Fig. 1c), likely reflecting the preference of reproductive males to move quickly around the edge of the pond and the preference of all crayfish to move quickly through the center of the pond.

To our knowledge, this is the first study to examine freshwater aquatic invertebrates using acoustic telemetry and high-dimensional positional data. This study was conducted in a small pond with limited habitat variability allowing for full receiver coverage and few obstacles to signal detection. However, these conditions also posed a challenge to data analysis due to the many resulting echoes that needed to be filtered. In detecting and positioning animals with acoustic telemetry, each location of an individual contains some amount of error. Therefore, for small, slow-moving organisms like RSC, increasingly short interpulse intervals can overestimate movement distance between each point. Transmitters in this study were set to have interpulse intervals between 6 and7 seconds, but our findings indicate that an interpulse interval around 30–120 s would likely provide adequate positional data resolution for crayfish based on the calculated distances moved. Adapting the methods and analyses in this study to other habitats will require a few considerations. For one, high-dimensional acoustic telemetry in riverine systems will likely need to confront greater habitat variability and acoustic noise that can pose challenges for detection and positioning^41^. Additionally, the RSC in this study belonged to a relatively closed population that were constrained to the telemetry array by environmental obstacles. In open freshwater systems, such as streams, RSC movements may not be limited to the telemetry array. In these cases, researchers may need to analyze movement “bursts” by individuals through the array and focus on measures of movement that apply on short timescales like movement steps, behavioral states, and occurrence distributions rather than range distributions^42^.

Our data were limited to the summer months in a temperate region, and we were unable to determine how movement patterns change when RSC are not reproductively active. Future research might seek to understand seasonal variation in movement patterns using these methods. Finally, RSC burrowing behavior has been well studied and is an important aspect of their biology^23,43^. They are also known to disperse overland^44^. While we were unable to attribute gaps in our data to either of these behaviors based on the data alone, this possibility remains. Future studies might seek to characterize, through observation or telemetry, when tagged crayfish leave the pond or burrow to help identify this signature in the acoustic telemetry data. This could then be applied to future data sets to reliably identify patterns of burrowing activity.

One of the reasons for the successful spread of RSC across the globe is their ability to inhabit many kinds of environments^19,20^. While our findings are limited to a single pond in their invasive range, our study provides a model for identifying movement patterns of RSC in other habitats (e.g. streams, canals), contexts (e.g. native range, invasion front) or management scenarios (e.g. post-treatment, trapping) and the ability to compare across studies. We examined RSC movement patterns with three different measures of movement, each with their own advantages. First, evaluating movement steps allowed us to see fine-scale patterns in the data. For example, we identified a spike in movement correlated with increased precipitation that isn’t seen in the other movement analyses we conducted (Fig. 2e). Second, analysis of movement states revealed differences in movement types between reproductive males and females that would have been hidden if we had only evaluated movement steps (Fig. 4b, c). This type of analysis is especially applicable to RSC where historical natural history observations suggested that two movement states are “real”^27–29^. Third, estimating range distribution emphasized a general pattern throughout the entire study and allowed us to identify the most important factor to RSC movement patterns – sex. Taken together, this study contributes to the understanding of RSC movement patterns, offers valuable data to inform management strategies, and provides future avenues of research aimed at mitigating the impacts of this globally invasive species.

## Methods

### Study site

**Fig. 6:**
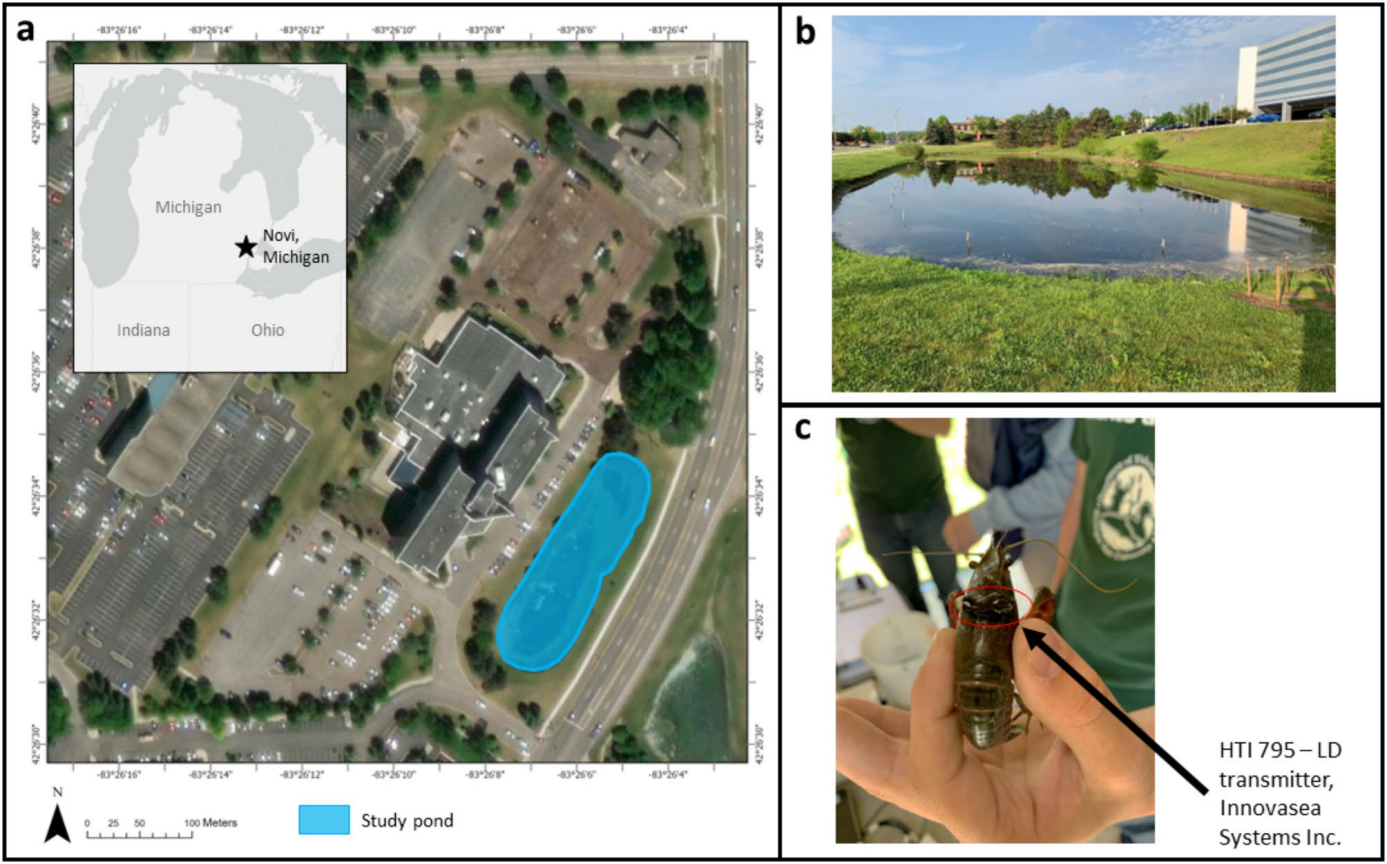
(**a**) Research location in Novi, Michigan, USA (base map generated with ArcGIS Pro software, version 3.4, https://pro.arcgis.com). (**b**) Image of research pond. (**c**) Red swamp crayfish (*Procambarus clarkii*) during transmitter application. Photo credit: Aaron Cupp.

Adult RSC were telemetered from May to July (2021) within an urban water retention pond near Novi, Michigan, USA (Fig. 6). The waterbody is ~ 2,500 cubic meters with a maximum depth of ~ 2 m [m], surface area of ~ 0.9 hectares, and functions as a water collection basin for stormwater runoff and flood mitigation^26^. An established population of RSC was first discovered at this site in 2017 through unknown origin and have since invaded several adjacent waterbodies and wetlands^45^. The study site was selected to better understand the movement patterns of RSC in a non-native range to help inform ongoing control efforts by state and federal resource management agencies. The pond had low overall aquatic species richness with few fish species present (channel catfish *Ictalurus punctatus*, green sunfish *Lepomis cyanellus*, yellow perch *Perca flavescens*, fathead minnow *Pimephales promelas*)^26^.

### Telemetry system and crayfish tagging

Red swamp crayfish positions were continuously monitored throughout the study period using a two-dimensional acoustic telemetry array. Sixteen individual hydrophones were positioned equidistantly throughout the pond and connected to a centralized acoustic telemetry receiver (Model 290, Hydroacoustic Telemetry System Incorporated HTI, Seattle, Washington, USA). Hydrophones were affixed to metal frames and submerged to ~ 0.1 m above the pond bottom. Each hydrophone was surveyed (Trimble^®^ RTK, Model R10, Sunnyvale, California, USA) for geospatial estimate calculations from individual transmitter signals.

Single HTI 795-LD transmitters (dimensions: 6.8 mm diameter, 20 mm length, 0.55 g wet weight, Innovasea Systems Inc., Boston, MA) were set to have pulse rates between 6,047 and 7,839 milliseconds [msec] and were attached to RSC at discrete tagging events on May 28, June 11, and June 18, 2021. Red swamp crayfish were collected from the study pond using Gee’s minnow traps (Tackle Factory^®^, Model G40M, 6.35-millimeter [mm] mesh) baited with approximately 150 g [g] of dry dog food in a small mesh bag. These harvest actions were part of an ongoing removal program to suppress invasive RSC populations by the state resource management agency. A subset of harvested RSC was allocated for acoustic telemetry observation (*N* = 24), and the remaining RSC were euthanized. The selection of RSC used for acoustic telemetry monitoring required that they were adult individuals in overall good health and had a sufficient body size (> 42 mm carapace length) to securely attach transmitters. We collected biological information for each tagged RSC including sex and carapace length. In addition, we identified morphotype of adult male RSC and took note if they were a reproductive form or type I male (M1) or a non-reproductive form or type II male (M2)^46^. Transmitters were externally attached to individual RSC by centrally bonding the transmitter to the carapace with a cyanoacrylate-based gel adhesive (Fig. 6c). Red swamp crayfish were held for approximately 10 min while the adhesive hardened and then released near the shorelines around the pond. Each RSC was visually monitored after release until they moved out of sight. Once in the water, acoustic transmitters submit signals at the specified pulse rate that are picked up by the receiver array. Signals recorded by receivers are post processed for identification from background noise and used to triangulate the position of the crayfish wearing that transmitter^14^.

### Telemetry data processing

More than 11 million total locations were collected from 24 individuals throughout May to July 2021. Individuals consisted of reproductive males (M1; *N* = 10), non-reproductive males (M2; *N* = 3), and females (F; *N* = 11). Red swamp crayfish carapace length ranged from 42 mm to 53 mm (mean = 47.73 mm; SD = 3.06 mm) (Table S6).

We visually inspected the data with the *adehabitatLT* v0.3.27 package in R v4.4.1^47,48^ and identified that the original raw positional data contained erroneous detections due to acoustic echoes in the pond. Location errors were clustered closely in time and space so that they resisted traditional filters by distance, angle, or velocity. We applied a coarse filter that calculated a rolling mean distance across 11 consecutive points and filtered all points with an average mean greater than 2 m to remove most of the clustered errors. We chose 2 m as a conservative estimate of the maximum distance a crayfish would move per minute based on previous studies^27–29^. We then manually filtered the remaining erroneous points based on visual inspection. RSC are slow moving animals that usually move small distances relative to our signal rate. Therefore, the remaining data sets contained jitteriness that could bias distance and speed estimates. This was resolved using a median smoothed data filter in the *altastools* package v1.0.0 in R v4.4.1 and a moving window (*K*) of 21^48,49^.

The final dataset for analysis excluded the first 24 h of data following RSC tagging to allow an acclimation period and was terminated on July 19th, 2021, immediately before a pesticide treatment was experimentally applied in the pond that would have impacted movements and behaviors. After cleaning, smoothing, and screening, our dataset included a total of 804,914 locations and monitoring times of RSC individuals ranged from 6.00 to 37.66 days (mean = 26.79 days, SD = 9.92 days).

We obtained mean distance from the pond edge for each hour by creating a spatial object representing the pond based on a survey of the pond edge (Trimble^®^ RTK, Model R10, Sunnyvale, California, USA). We then calculated distance from the edge for each location and averaged them for each hour using the st_distance function of *sf* package v1.0.17 in R v4.4.1^48,50^. Hourly air temperature (°C) and precipitation data (mm) were obtained for the study location from the 1/8th degree meteorological forcing dataset for Phase 2 of the North American Land Data Assimilation System (NLDAS-2)^51^.

### Measures of movement

We assessed movement at three scales: movement steps, overall space-use (range distribution), and behavioral states. First, we measured movement steps as the total distance moved per hour for each crayfish. Next, we estimated the range distribution of crayfish across the entire study period as a measure of overall space-use. We used a weighted autocorrelated kernel density estimation (wAKDE) in the *ctmm* package v1.2.0 in R v4.4.1 to estimate the 50% and 95% range distribution areas^48,52^. These values represent the maximum likelihood area where an animal spends 50% and 95% of their time and indicate a “core area,” or the median area, and broader range distribution, respectively. Fine-scale acoustic telemetry data is inherently spatially autocorrelated and often contains gaps when animals leave the array area or cannot be detected due to other factors. The wAKDE accounts for spatial autocorrelation by appropriately weighting under-sampled and over-sampled areas, allowing for improved projection of future space-use by individual animals^53^. To fit the wAKDE, we first used AICc-based model selection to identify the continuous-time movement model that best represented our data, including the Independent and Identically Distributed process, the Ornstein-Uhlenbeck (OU) process, and the OU-Foraging process^52,53^. We then estimated 50% and 95% range distributions within the boundaries of the pond based on the best-fit movement model for each individual^52,54^.

Finally, we describe the behavioral states of RSC using hidden Markov models (HMMs). HMMs allowed us to classify crayfish detection locations into the latent movement patterns that may have given rise to our observed data. Our models were informed using the step lengths (distance traveled [m] per unit time) and turning angles (change in direction [radians] from time *t* to time *t* + 1) of tagged RSC. We estimated the parameters of the HMM using the *momentuHMM* package v1.5.5 in R v4.4.1^48,55^. HMMs require a regularly spaced time series to model movement states^42^. Therefore, we first fit a correlated random walk (CRW) model to our cleaned, smoothed, and irregular RSC locations using the *crawlWrap* function^48,55^. The CRW estimated discrete crayfish locations (latitude and longitude) every hour to limit autocorrelation within the data. Our CRW models were then used to fit a two-state HMM. We fit two states, rather than models with three or more states, because a limited understanding of fine-scale behavioral movements of crayfish in the literature did not justify the use of more complex models^56^.

We modeled the transition probabilities between movement states as well as the step length and turning angle distribution parameters with the *fitHMM* function^48,55^. Step lengths were assumed to follow a gamma distribution and turning angles were modeled using a wrapped Cauchy distribution^55^. State transition probabilities and mean step length were modeled as a function of sex, carapace length (mm), time of day (0–24), air temperature (°C), and distance from the pond edge (m). We included sex as a factor having three levels (M1, M2, F), air temperature and distance from the pond edge as continuous covariates, and time of day as a cyclic covariate. Cyclic covariates are estimated with two periodic functions, cos($\:\frac{2\pi\:t}{24}$) and sin($\:\frac{2\pi\:t}{24}$), where *t* is the time of day (hour; 0–24) and 24 is the assumed daily periodicity of the function^55,57^. We performed model selection using AIC values and chose the model with the lowest AIC value as the true model^58^. We assessed model fit by examining bivariate and quantile plots of the pseudo residuals (Fig. S1.). Lastly, we explored the estimated probabilities of remaining in a movement state given some fixed value of a covariate and estimated the effect of covariates on the mean step lengths of RSC using the Viterbi algorithm^55^.

### Statistical analyses

We examined the effect of individual and environmental factors on movement steps and estimated range distribution (50% and 95% wAKDE) of invasive RSC crayfish with two quantitative approaches. First, we applied a generalized additive model (GAM) with a gamma distribution and log-link function to test the non-linear effects of sex, hour of day, hour of year, temperature, precipitation, and distance to the pond edge on distance per hour. We selected a gamma distribution based on the distribution of our movement data, which was continuous, positive, and right-skewed. We included sex as a factor having three levels (M1, M2, F), hour of year (0-8760) as a cubic spline, hour of day (0–24) as a cyclic spline, temperature, precipitation, and distance to the pond edge were continuous covariates with non-linear relationships to our response variable so were included as smooth terms. We also included the interaction of individual by hour of year as a factor smoothed random effect to account for differences in the length and period of data collection for each individual. We included the full model in our results. Second, we used a generalized linear model (GLM) with a gamma distribution to understand the parameters contributing to range distribution differences, including sex, carapace length (mm), and length of data collection (days) on 50% and 95% range distribution estimates (wAKDE). We selected a gamma distribution based on the distribution of our data. We included sex as a factor having three levels (M1, M2, F), and carapace length and length of data collection as continuous covariates. We conducted stepwise model selection with AICc and considered the model with the lowest value as the best fit model^58^. All analyses were conducted with the *mgcv* package v1.9.1 in R v4.4.1^48,59^.

## Electronic supplementary material

Below is the link to the electronic supplementary material.

Supplementary Material 1

## Data Availability

All data are publicly available through ScienceBase at 10.5066/P92OOTED.

## References

[1] Reid, A. J. et al. Emerging threats and persistent conservation challenges for freshwater biodiversity. *Biol. Rev.* **94**, 849–873 (2019). 30467930 10.1111/brv.12480

[2] Strayer, D. L. & Dudgeon, D. Freshwater biodiversity conservation: recent progress and future challenges. *J. North. Am. Benthol Soc.* **29**, 344–358 (2010).

[3] Almond, R., Grooten, M., Bignoli, D. J. & Petersen, T. *Living Planet Report 2022—Building a Nature—Positive Society*. (2022).

[4] Strayer, D. L. Alien species in fresh waters: ecological effects, interactions with other stressors, and prospects for the future. *Freshw. Biol.* **55**, 152–174 (2010).

[5] Gallardo, B., Clavero, M., Sánchez, M. I. & Vilà, M. Global ecological impacts of invasive species in aquatic ecosystems. *Glob Change Biol.* **22**, 151–163 (2016). 10.1111/gcb.13004 26212892

[6] Lennox, R. J. et al. Envisioning the future of aquatic animal tracking: technology, science, and application. *BioScience* **67**, 884–896 (2017).

[7] Matley, J. K. et al. Global trends in aquatic animal tracking with acoustic telemetry. *Trends Ecol. Evol.* **37**, 79–94 (2022). 34563403 10.1016/j.tree.2021.09.001

[8] Meira, A., Carvalho, F., Castro, P. & Sousa, R. Applications of biosensors to overcome monitoring challenges in freshwater invasive species. *Preprint At.*10.3897/arphapreprints.e129002 (2024).

[9] Allen, A. M. & Singh, N. J. Linking movement ecology with wildlife management and conservation. *Front. Ecol. Evol.* **3** (2016).

[10] Nathan, R. et al. A movement ecology paradigm for unifying organismal movement research. *Proc. Natl. Acad. Sci.* **105**, 19052–19059 (2008). 19060196 10.1073/pnas.0800375105 PMC2614714

[11] Kay, S. L. et al. Quantifying drivers of wild pig movement across multiple spatial and temporal scales. *Mov. Ecol.* **5**, 14 (2017). 28630712 10.1186/s40462-017-0105-1 PMC5471724

[12] Bergman, J. N. et al. Tracking the early stages of an invasion with biotelemetry: behaviour of round goby (*Neogobius melanostomus*) in Canada’s historic Rideau Canal. *Biol. Invasions*. **24**, 1149–1173 (2022).

[13] Dahl, K. A. & Patterson, W. F. Movement, home range, and depredation of invasive Lionfish revealed by fine-scale acoustic telemetry in the Northern Gulf of Mexico. *Mar. Biol.* **167**, 111 (2020).

[14] Raboin, M. et al. Movement and behavioral States of common carp (*Cyprinus carpio*) in response to a behavioral deterrent in a navigational lock. *Mov. Ecol.* **11**, 42 (2023). 37496021 10.1186/s40462-023-00396-z PMC10373248

[15] Spiegel, O., Leu, S. T., Bull, C. M. & Sih, A. What’s your move? Movement as a link between personality and Spatial dynamics in animal populations. *Ecol. Lett.* **20**, 3–18 (2017). 28000433 10.1111/ele.12708

[16] Daniels, J. A. & Kemp, P. S. Personality-dependent passage behaviour of an aquatic invasive species at a barrier to dispersal. *Anim. Behav.* **192**, 63–74 (2022).

[17] Boback, S. M., Nafus, M. G., Yackel Adams, A. A. & Reed, R. N. Invasive brown treesnakes (*Boiga irregularis*) move short distances and have small activity areas in a high prey environment. *Sci. Rep.* **12**, 12705 (2022). 35882893 10.1038/s41598-022-16660-y PMC9325984

[18] Oficialdegui, F. J., Sánchez, M. I. & Clavero, M. One century away from home: how the red swamp crayfish took over the world. *Rev. Fish. Biol. Fish.* **30**, 121–135 (2020).

[19] Gherardi, F. Crayfish invading Europe: the case study of *Procambarus Clarkii*. *Mar. Freshw. Behav. Physiol.* **39**, 175–191 (2006).

[20] Loureiro, T. G., Anastácio, P. M. S. G., Araujo, P. B. & Souty-Grosset, C. Almerão, M. P. Red swamp crayfish: biology, ecology and invasion - an overview. *Nauplius* **23**, 1–19 (2015).

[21] Loureiro, T. G., Anastácio, P. M., Bueno, S. L. D. S., Wood, C. T. & Araujo, P. B. Food matters: trophodynamics and the role of diet in the invasion success of *Procambarus Clarkii* in an Atlantic forest conservation area. *Limnologica* **79**, 125717 (2019).

[22] Jussila, J., Vrezec, A., Makkonen, J., Kortet, R. & Kokko, H. Invasive crayfish and their invasive diseases in Europe with the focus on the virulence evolution of the crayfish plague. in *Biological Invasions in Changing Ecosystems* (ed. Canning-Clode, J.) 183–211 (De Gruyter Open, 2015). 10.1515/9783110438666-013

[23] Barbaresi, S., Tricarico, E. & Gherardi, F. Factors inducing the intense burrowing activity of the red-swamp crayfish, *Procambarus clarkii*, an invasive species. *Naturwissenschaften* **91** (2004). 10.1007/s00114-004-0533-9 15257390

[24] Budnick, W. et al. Evaluation of five trap designs for removal of invasive red swamp crayfish (*Procambarus Clarkii* Girard, 1852) in Southern Michigan: catch per unit effort, body size, and sex biases. *Manag Biol. Invasions*. **13**, 369–390 (2022).

[25] Fredricks, K., Tix, J., Smerud, J. & Cupp, A. Laboratory trials to evaluate carbon dioxide as a potential behavioral control method for invasive red swamp (*Procambarus clarkii*) and Rusty crayfish (*Faxonius rusticus*). *Manag Biol. Invasions*. **11**, 259–278 (2020).

[26] Smerud, J. et al. Field application of carbon dioxide as a behavioral control method for invasive red swamp crayfish (*Procambarus Carkii*) in Southeastern Michigan Water Retention. *U. S. Geological Survey Open-File Report*. (2022).

[27] Barbaresi, S., Santini, G., Tricarico, E. & Gherardi, F. Ranging behaviour of the invasive crayfish, *Procambarus Clarkii* (Girard). *J. Nat. Hist.* **38**, 2821–2832 (2004).

[28] Anastácio, P. M. et al. Indicators of movement and space use for two co-occurring invasive crayfish species. *Ecol. Indic.* **53**, 171–181 (2015).

[29] Gherardi, F., Barbaresi, S. & Salvi, G. Spatial and Temporal patterns in the movement of *Procambarus clarkii*, an invasive crayfish. *Aquat. Sci.* **62**, 179–193 (2000).

[30] Hussey, N. E. et al. Aquatic animal telemetry: A panoramic window into the underwater world. *Science* **348**, 1255642 (2015). 26068859 10.1126/science.1255642

[31] Cupp, A. R. et al. Acoustic telemetry evaluation of invasive red swamp crayfish (Procambarus clarkii) behavior in Southern Michigan (summer 2021). *U S Geol. Surv.*10.5066/P92OOTED (2023).

[32] Alcorlo, P., Geiger, W. & Otero, M. Reproductive biology and life cycle of the invasive crayfish *Procambarus Clarkii* (Crustacea: Decapoda) in diverse aquatic habitats of South-Western Spain: implications for population control. *Fundam Appl. Limnol.* **173**, 197–212 (2008).

[33] Hamasaki, K., Dan, S. & Kawai, T. Reproductive biology of the red swamp crayfish *Procambarus Clarkii* (Girard, 1852) (Decapoda: astacidea: Cambaridae): A review. *J. Crustac Biol.* **43**, ruad057 (2023).

[34] Baena, M. L. & Macías-Ordóñez, R. Mobility and mating frequency in the scramble competition polygyny of a chrysomelid beetle. *Behav. Ecol.* **26**, 416–424 (2015).

[35] Rubenstein, D. R. *Animal Behavior* (Sinauer Associates/Oxford University, 2023).

[36] Chucholl, C. Population ecology of an alien warm water crayfish (*Procambarus clarkii*) in a new cold habitat. *Knowl. Manag Aquat. Ecosyst.* **29**. 10.1051/kmae/2011053 (2011).

[37] Peruzza, L. et al. Reproductive plasticity of a *Procambarus Clarkii* population living 10°C below its thermal optimum. *Aquat. Invasions*. **10**, 199–208 (2015).

[38] Wutz, S. & Geist, J. Sex- and size-specific migration patterns and habitat preferences of invasive signal crayfish (*Pacifastacus Leniusculus* Dana). *Limnologica* **43**, 59–66 (2013).

[39] Gherardi, F., Tricarico, E. & Ilhéu, M. Movement patterns of an invasive crayfish, *Procambarus clarkii*, in a temporary stream of Southern Portugal. *Ethol. Ecol. Evol.* **14**, 183–197 (2002).

[40] Musil, M., Buric, M., Policar, T., Kouba, A. & Kozak, P. Comparison of diurnal and nocturnal activity between noble crayfish (*Astacus astacus*) and spinycheek crayfish (*Orconectes limosus*). *Freshw. Crayfish*. **17**, 189–193 (2010).

[41] Bergé, J. et al. Probability of detection and positioning error of a hydro acoustic telemetry system in a fast-flowing river: intrinsic and environmental determinants. *Fish. Res.* **125–126**, 1–13 (2012).

[42] Alston, J. M. et al. Clarifying space use concepts in ecology: range vs. occurrence distributions. (2022). 10.1101/2022.09.29.509951 10.1002/ecy.70300 PMC12966954 41793168

[43] Correia, A. M. & Ferreira, O. Burrowing behavior of the introduced red swamp crayfish *Procambarus Clarkii* (Decapoda: Cambaridae) in Portugal. *J. Crustac Biol.* **15**, 248–257 (1995).

[44] Thomas, J. R. et al. Terrestrial emigration behaviour of two invasive crayfish species. *Behav. Processes*. **167**, 103917 (2019). 31349024 10.1016/j.beproc.2019.103917

[45] Smith, K. et al. Assessment of invasion risks for red swamp crayfish (*Procambarus clarkii*) in Michigan, USA. *Manag Biol. Invasions*. **9**, 405–415 (2018).

[46] Hamasaki, K., Osabe, N., Nishimoto, S., Dan, S. & Kitada, S. Sexual dimorphism and reproductive status of the red swamp crayfish *Procambarus Clarkii*. *Zool. Stud.* **59** (2020). 10.6620/ZS.2020.59-07 PMC7396923 32760453

[47] Calenge, C., Dray, S. & Royer, M. adehabitatLT: Analysis of animal movements. (2023).

[48] R Core Team. *R: A Language and Environment for Statistical Computing* (R Foundation for Statistical Computing, 2024).

[49] Gupte, P. R. et al. A guide to pre-processing high‐throughput animal tracking data. *J. Anim. Ecol.* **91**, 287–307 (2022). 34657296 10.1111/1365-2656.13610 PMC9299236

[50] Pebesma, E. Simple features for R: Standardized support for spatial vector data. 10, (2018).

[51] Xia, Y. et al. Continental-scale water and energy flux analysis and validation for the North American land data assimilation system project phase 2 (NLDAS-2): 1. Intercomparison and application of model products. *J. Geophys. Res. Atmos.* **117** (2012).

[52] Calabrese, J. M., Fleming, C. H. & Gurarie, E. Ctmm: an R package for analyzing animal relocation data as a continuous-time stochastic process. *Methods Ecol. Evol.* **7**, 1124–1132 (2016).

[53] Silva, I. et al. Autocorrelation-informed home range estimation: A review and practical guide. *Methods Ecol. Evol.* **13**, 534–544 (2022).

[54] Fleming, C. H. & Calabrese, J. M. A new kernel density estimator for accurate home-range and species‐range area Estimation. *Methods Ecol. Evol.* **8**, 571–579 (2017).

[55] McClintock, B. T., Michelot, T. & momentuHMM: R package for generalized hidden Markov models of animal movement. *Methods Ecol. Evol.* **9**, 1518–1530 (2018).

[56] Pohle, J., Langrock, R., Van Beest, F. M. & Schmidt, N. M. Selecting the number of States in hidden Markov models: pragmatic solutions illustrated using animal movement. *J. Agric. Biol. Environ. Stat.* **22**, 270–293 (2017).

[57] Bacheler, N. M., Michelot, T., Cheshire, R. T. & Shertzer, K. W. Fine-scale movement patterns and behavioral States of Gray triggerfish *Balistes Capriscus* determined from acoustic telemetry and hidden Markov models. *Fish. Res.* **215**, 76–89 (2019).

[58] Burnham, K. P. & Anderson, D. R. *Model Selection and Mulitmodel Inference: A Practical Information-Theoretic Approach* (Springer, 2002).

[59] Wood, S. N. Fast stable restricted maximum likelihood and marginal likelihood Estimation of semiparametric generalized linear models. *J. R Stat. Soc. Ser. B Stat. Methodol.* **73**, 3–36 (2011).

