# Supplementary material for: Movement patterns of invasive red swamp crayfish vary with sex and environmental factors
Source: Sci Rep. 2025 Apr 24;15:14312. doi: 10.1038/s41598-025-96379-8 (PMC12022145; doi:10.1038/s41598-025-96379-8)
Supplement: Supplementary file 1 — Supplementary Material 1 [file 41598_2025_96379_MOESM1_ESM.docx]

TITLE

Supplementary file: Movement patterns of invasive red swamp crayfish (*Procambarus clarkii*) vary with sex and environmental factors.

AUTHORS

Maggie Raboin^1*^, Brian M. Roth^2^, Aaron Sullivan^2^, Ann L. Allert^3^, Jim A. Stoeckel^4^, Lucas R. Nathan^5^, Kathleen B. Quebedeaux^5^, Matthew D. Sholtis^1^, Justin R. Smerud^6^, Richard A. Erickson^6^, and Aaron R. Cupp^6^

*Author Details:*

*^1^U.S. Geological Survey, Columbia River Research Laboratory, 5501A Cook-Underwood Rd, Cook, Washington, USA 98605.*

*^2^Department of Wildlife and Fisheries, Michigan State University, East Lansing, MI, USA.*

*^3^U.S. Geological Survey, Columbia Environmental Research Center, 4200 New Haven Road
Columbia, Missouri, USA 65201.*

*^4^School of Fisheries, Aquaculture, and Aquatic Sciences, Auburn University, Auburn, Alabama, USA.*

*^5^* *Michigan Department of Natural Resources, Lansing, MI, USA.*

*^6^U.S. Geological Survey, Upper Midwest Environmental Sciences Center, 2630 Fanta Reed Rd., La Crosse, Wisconsin, USA 54603.*

** Corresponding author: mraboin@usgs.gov*

**Table S1.** ⎯ Summary of full generalized additive model (GAM) results for movement steps (distance per hour) of invasive red swamp crayfish (*Procambarus clarkii*). Predictor variables included sex (reproductive male [M1], non-reproductive male [M2], and female [F]), precipitation (precip), distance to pond edge (edge), hour of year (hoy), hour of day (time), temperature (temp), and crayfish id (id). All continuous predictor variables were fit as smoothing parameters.

| **Family** | **Link Function** | **Formula** | | |
| --- | --- | --- | --- | --- |
| Gamma | Logit | Dist ~ sex + s(precip) + s(edge) + s(hoy, k=100, bs=”cr”) + s(time, k=24, bs=”cc”) + s(temp) + s(id, hoy, bs=”fs”) | | |
|  | | | | |
| **Parametric coefficients** | | | | |
|  | **Estimate** | **Std. Error** | ***z-*value** | **Pr(> \|z\|)** |
| (Intercept) | 0.997 | 0.232 | 4.30 | <0.001 |
| sex M2 | -0.823 | 0.473 | -1.74 | 0.082 |
| sex F | -0.750 | 0.309 | -2.43 | 0.015 |
|  | | | | |
| **Approximate Significance of Smooth Terms** | | | | |
|  | **Estimated df** | **χ^2^** | ***p*-value** |  |
| s(precip) | 1.742 | 0.823 | 0.411 |  |
| s(edge) | 8.783 | 30.666 | <0.001 |  |
| s(hoy) | 85.885 | 7.328 | <0.001 |  |
| s(time) | 16.580 | 20.312 | <0.001 |  |
| s(temp) | 3.899 | 3.300 | 0.006 |  |
| s(id, hoy) | 138.691 | 20.520 | <0.001 |  |
|  |  |  |  |  |
| **Adjusted R2** | **Deviance Explained** |  |  |  |
| 0.357 | 54.9% |  |  |  |

**Table S2.** ⎯ Model selection table for 95% and 50% range distribution estimate. The model fit as indicated by degrees of freedom (df), corrected Akaike’s Information Criteria (AICc), and difference in AICc between a given model and the top model (ΔAICc) are given. Predictor variables included sex (reproductive male [M1], non-reproductive male [M2], and female [F]), carapace length (carapace), and number of days of data collection for each individual (days).

| Model | df | AICc | ΔAICc |
| --- | --- | --- | --- |
| *95% HR models* |  |  |  |
| sex | 4 | 378.63 | 0 |
| days | 3 | 380.12 | 1.49 |
| carapace | 3 | 380.39 | 1.76 |
| sex + days | 5 | 381.00 | 2.37 |
| sex + carapace | 5 | 381.50 | 2.87 |
| days + carapace | 4 | 382.93 | 4.30 |
| sex + days + carapace | 6 | 384.55 | 5.92 |
|  |  |  |  |
| *50% HR models* |  |  |  |
| sex | 4 | 312.47 | 0 |
| sex + days | 5 | 315.39 | 2.92 |
| sex + carapace | 5 | 315.70 | 3.23 |
| days | 3 | 318.61 | 6.14 |
| carapace | 3 | 318.84 | 6.37 |
| sex + days + carapace | 6 | 318.96 | 6.49 |
| days + carapace | 4 | 321.51 | 9.04 |

**Table S3.** ⎯ Summary of best fit generalized linear model results for 95% and 50% range distribution estimates.

| 95% HR model results | | | |
| --- | --- | --- | --- |
| *Predictors* | *Estimates (m^2^)* | *Conf. Interval (m^2^)* | *P* |
| Intercept (M1) | 1687.86 | 917.23 – 3628.20 | <0.001 |
| M2 male | 556.99 | 151.91 – 2920.00 | 0.122 |
| Female | 523.24 | 202.54 – 1333.41 | 0.013 |
|  |  |  |  |
| 50% HR model results | | | |
| *Predictors* | *Estimates* | *Conf. Interval (m^2^)* | *P* |
| Intercept (M1) | 636.23 | 311.39 – 1623.93 | <0.001 |
| M2 male | 101.80 | 25.45 – 827.10 | 0.036 |
| Female | 108.16 | 31.81 – 337.20 | 0.002 |

**Table S4.** ⎯ Model selection results for red swamp crayfish (*Procambarus clarkii*) HMM *momentuHMM* movement models. The model fit as indicated by Akaike’s Information Criteria (AIC), and difference in AIC between a given model and the top model (ΔAIC) are given. Predictor variables included sex (reproductive male [M1], non-reproductive male [M2], and female [F]), hour of day (time), distance to the pond edge (edge), temperature (temp), and carapace length (carapace).

| Model | AIC | ∆AIC |
| --- | --- | --- |
| sex + time + edge | 65977.80 | 0.00 |
| sex + time + edge + temp | 65979.74 | 1.94 |
| sex + time | 65996.34 | 18.54 |
| sex + time + temp | 65997.36 | 19.56 |
| sex + temp | 66092.82 | 115.02 |
| sex + edge | 66097.85 | 120.05 |
| sex | 66125.80 | 148.00 |
| time | 66133.02 | 155.22 |
| edge | 66189.53 | 211.73 |
| temp | 66222.27 | 244.47 |
| carapace | 66249.69 | 271.89 |
| null | 66268.61 | 290.81 |


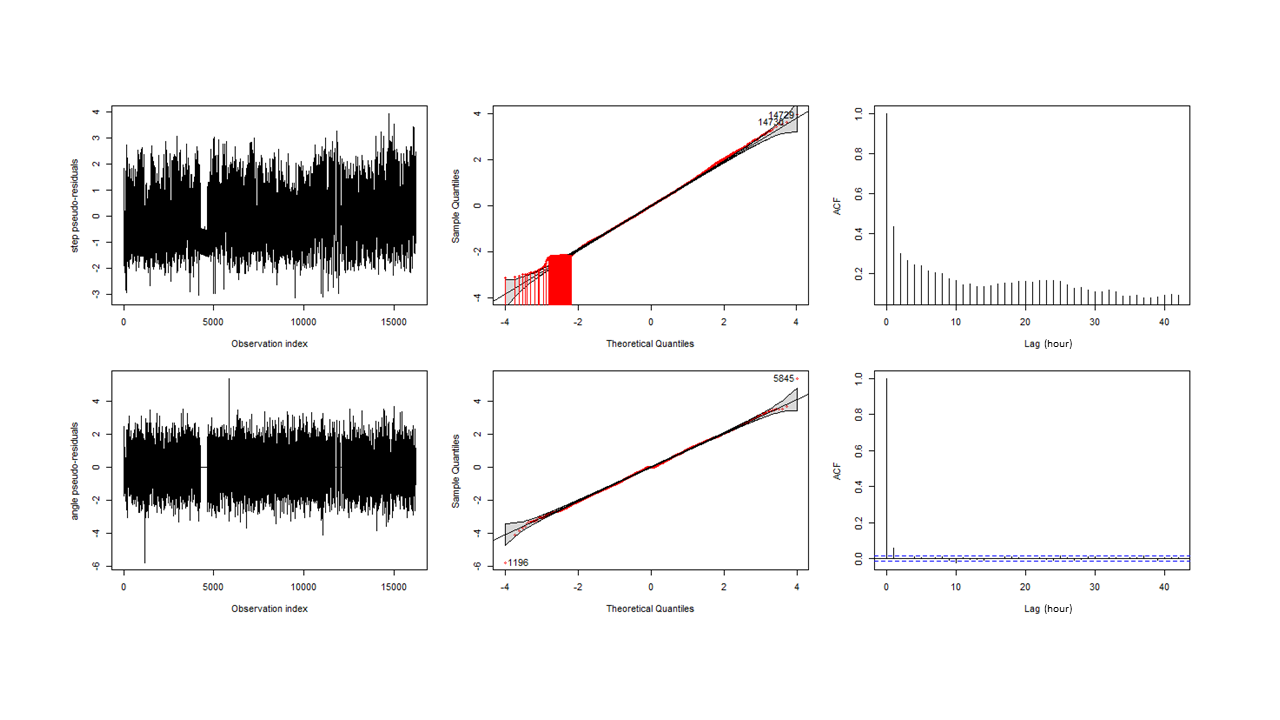


**Figure S1.** ⎯ Pseudo-residual plots of time-series, QQ plots, and sample temporal autocorrelation function for red swamp crayfish (*Procambarus clarkii*) *momentuHMM* movement models.


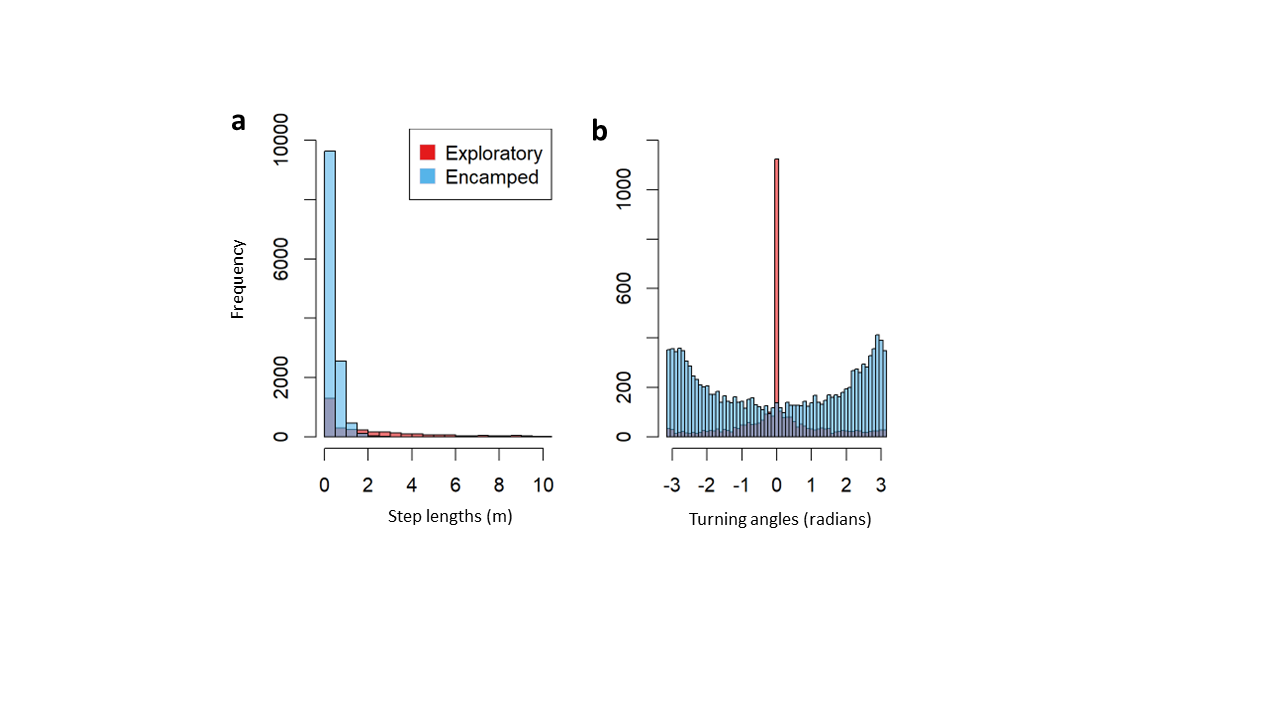


**Figure S2.** ⎯ Distribution of (a) step lengths and (b) turning angles by the modeled behavioral states.

**Table S5.** ⎯ Coefficients for effects of covariates on the transition probabilities and mean step lengths of red swamp crayfish (*Procambarus clarkii*). Predictor variables included sex (reproductive male [M1], non-reproductive male [M2], and female [F]), hour of day (time), and distance to the pond edge (edge).

| Parameter | Mean step coefficient | | Transition probabilities | |
| --- | --- | --- | --- | --- |
|  | Encamped | Exploratory | Encamped -> Exploratory | Exploratory -> Encamped |
| *Intercept* | -0.99 | 0.83 | -3.00 | -2.96 |
| Sex F | -0.02 | -0.15 | -1.10 | 0.62 |
| Sex M2 | -0.00 | -0.24 | -1.38 | 0.64 |
| Edge | 0.01 | 0.06 | -0.02 | 0.08 |
| CosinorCos(time) | -0.05 | 0.03 | -0.81 | 0.36 |
| CosinorSin(time) | -0.04 | -0.22 | -0.54 | 0.21 |
| Std. deviation | -1.25 | 1.83 |  |  |
| Zero mass | -4.20 | -6.76 |  |  |

**Table S6.** ⎯ Information for individual red swamp crayfish (*Procambarus clarkii*) used for telemetry observation in Michigan, USA in 2021. Individuals consisted of reproductive males (M1), non-reproductive males (M2), and females (F). All data are publicly available through ScienceBase at https://doi.org/10.5066/P92OOTED^1^.

| Tag ID | Sex/Form | Carapace  length (mm) | Dates | Number of days | Number of estimated positions |
| --- | --- | --- | --- | --- | --- |
| 6047 | F | 42 | 5/28 – 7/01 | 33.22 | 36,601 |
| 6094 | F | 53 | 6/11 – 7/12 | 30.83 | 38,916 |
| 6141 | M2 | 48 | 6/18 – 7/19 | 30.49 | 42,983 |
| 6235 | F | 49 | 5/28 – 6/17 | 20.21 | 22,753 |
| 6282 | M1 | 47 | 6/11 – 6/22 | 10.49 | 13,593 |
| 6329 | F | 42 | 6/18 – 7/19 | 30.64 | 41,601 |
| 6423 | F | 49 | 5/28 – 6/13 | 15.76 | 17,193 |
| 6470 | M1 | 43 | 6/11 – 6/21 | 10.22 | 20,487 |
| 6517 | M1 | 52 | 6/18 – 7/19 | 30.20 | 37,403 |
| 6611 | M2 | 50 | 5/28 – 6/26 | 28.88 | 36,878 |
| 6658 | M1 | 49 | 6/11 – 7/11 | 29.60 | 33,230 |
| 6705 | F | 46 | 6/18 – 7/19 | 30.63 | 41,567 |
| 6799 | M2 | 46 | 5/28 – 6/26 | 28.31 | 38,364 |
| 6847 | F | 49 | 6/11 – 7/19 | 37.66 | 47,268 |
| 6894 | M1 | 50 | 6/18 – 7/14 | 36.05 | 34,785 |
| 7036 | M1 | 49 | 6/11 – 7/14 | 37.25 | 49,054 |
| 7225 | M1 | 49 | 6/11 – 7/16 | 35.19 | 28,593 |
| 7272 | M1 | 47 | 6/18 – 7/02 | 14.12 | 15,992 |
| 7414 | F | 49 | 6/11 – 7/19 | 37.65 | 49,619 |
| 7461 | F | 42 | 6/18 – 7/19 | 30.63 | 43,113 |
| 7603 | M1 | 47 | 6/11 – 7/19 | 37.65 | 48,148 |
| 7650 | M1 | 48 | 6/18 – 6/24 | 6.00 | 6,836 |
| 7792 | F | 49 | 6/11 – 7/19 | 37.63 | 47,878 |
| 7839 | F | 53 | 6/18 – 7/02 | 13.66 | 12,059 |

REFERENCES

1. Cupp, A. R. *et al.* Acoustic telemetry evaluation of invasive red swamp crayfish (*Procambarus clarkii*) behavior in Southern Michigan (summer 2021). U.S. Geological Survey https://doi.org/10.5066/P92OOTED (2023).
